# Supplementary figures and images for: Physiology-informed regularisation enables training of universal differential equation systems for biological applications
Source: PLoS Comput Biol. 2025 Jan 23;21(1):e1012198. doi: 10.1371/journal.pcbi.1012198 (PMC11771921; doi:10.1371/journal.pcbi.1012198)

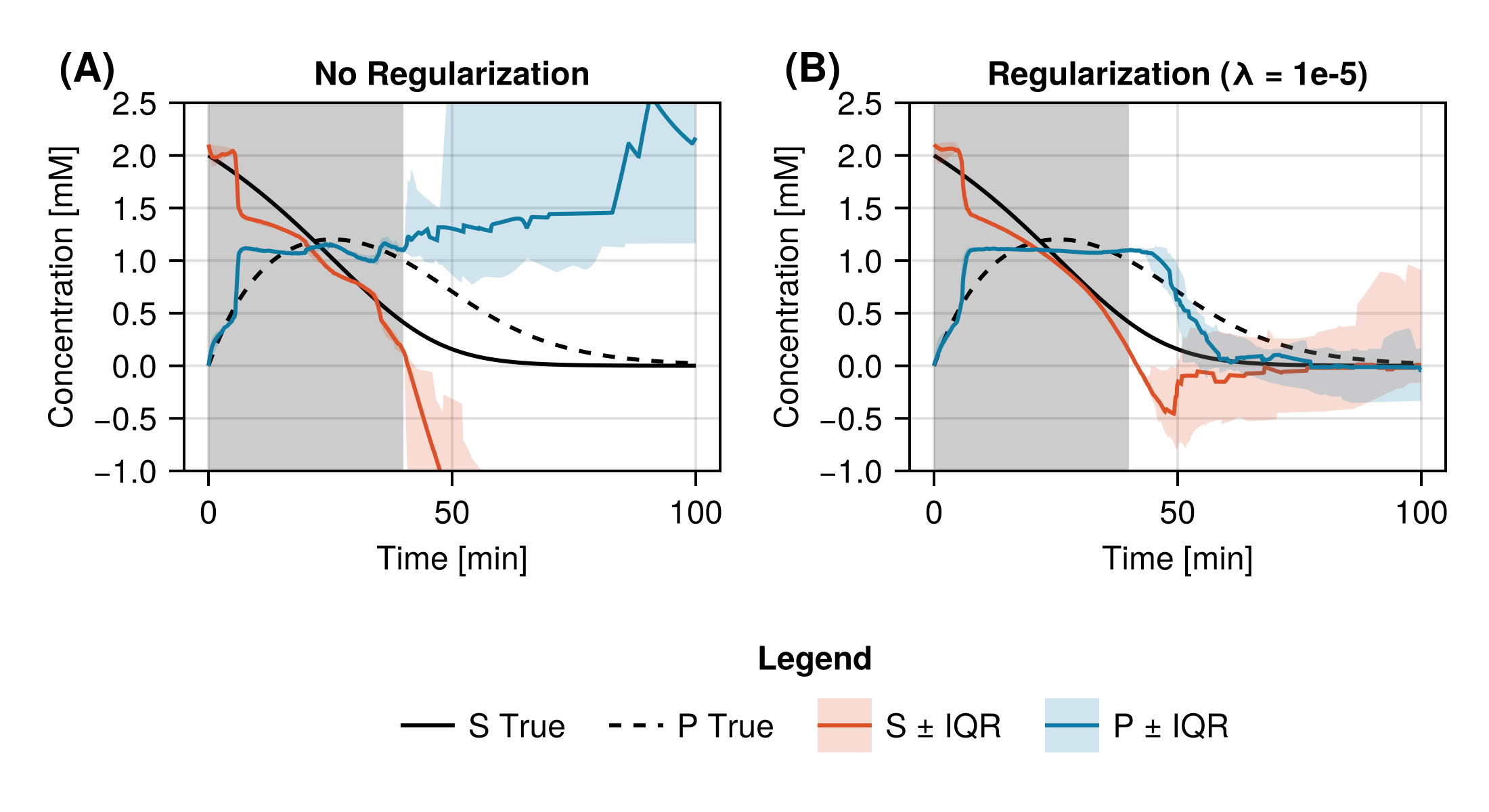

Supplement: S1 Fig — Median predictions for the Michealis-Menten model for species S (red) and species P (blue) including the first and third quartiles for A: the case without regularisation (λ = 0), and B: mild regularisation (λ = 10-5) for a sampling duration of 40 minutes. The red and blue shaded regions indicate the interquartile range of the top 25 models, selected based on the training error, for species S and P respectively. The sampling duration used for training is marked in the grey shaded region. The ground truth model is also visualised with the black solid (S) and dashed (P) lines to allow comparison of the model fits. (TIF) [file pcbi.1012198.s001.tif]

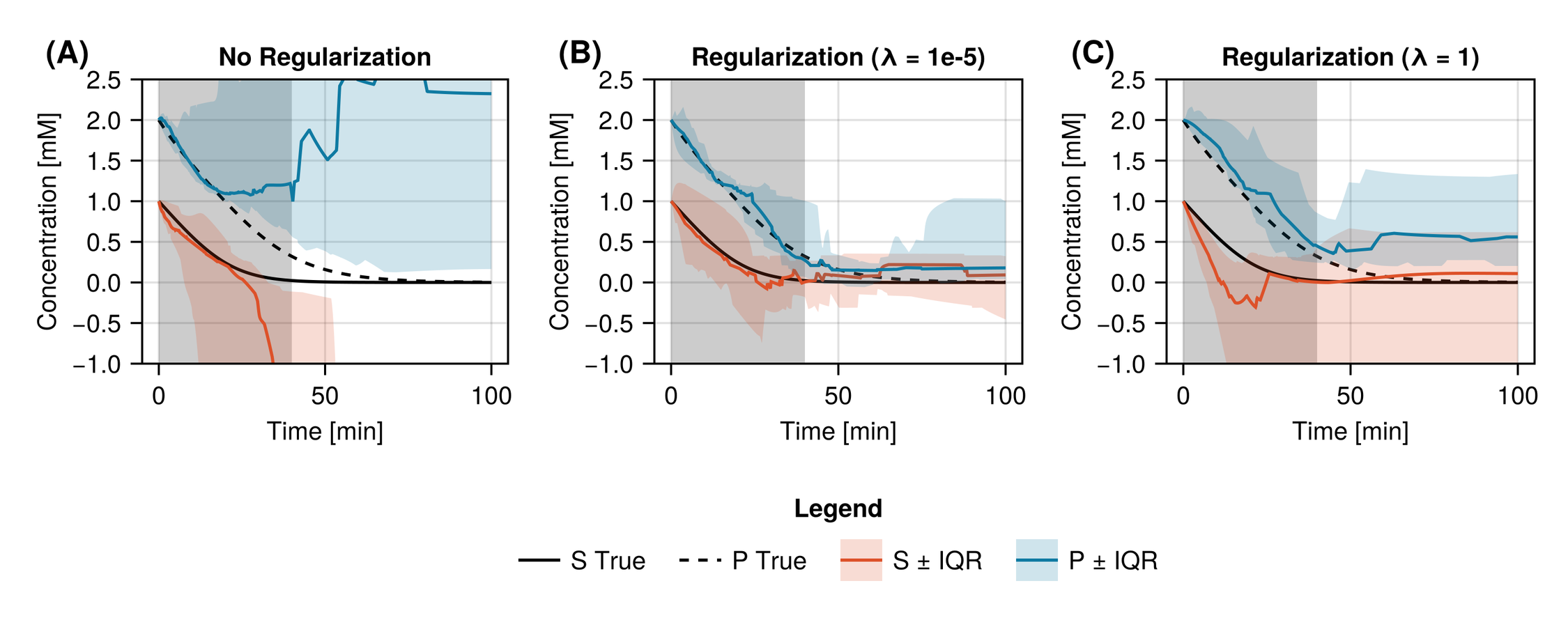

Supplement: S2 Fig — Median predictions for the Michealis-Menten model for species S (red) and species P (blue) including the first and third quartiles for A: the case without regularisation (λ = 0), B: mild regularisation (λ = 10-5), and C: strong regularisation (λ = 1) for a sampling duration of 40 minutes. The red and blue shaded regions indicate the interquartile range of the top 25 models, selected based on the training error, for species S and P respectively. The sampling duration used for training is marked in the grey shaded region. The ground truth model is also visualised with the black solid (S) and dashed (P) lines to allow comparison of the model fits. (TIF) [file pcbi.1012198.s002.tif]

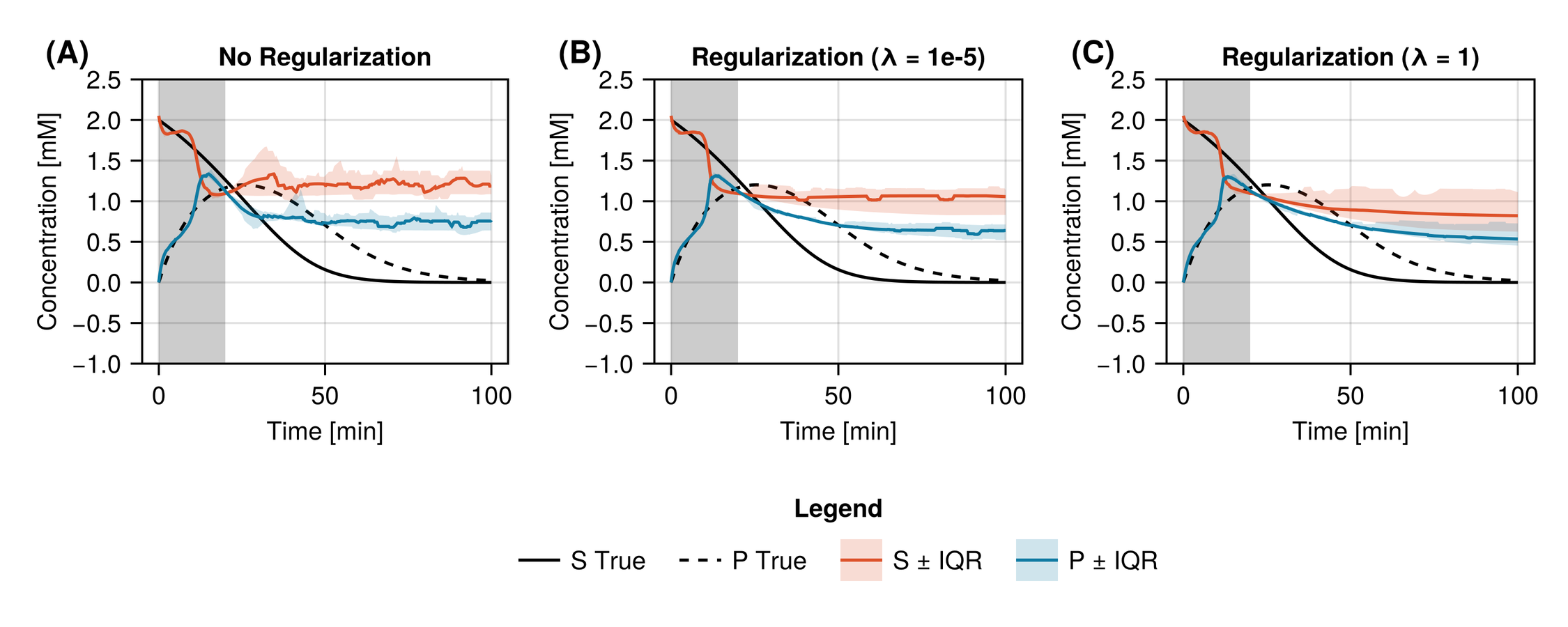

Supplement: S3 Fig — Median predictions for the Michealis-Menten model for species S (red) and species P (blue) including the first and third quartiles for A: the case without regularisation (λ = 0), B: mild regularisation (λ = 10-5), and C: strong regularisation (λ = 1) for a sampling duration of 20 minutes. The red and blue shaded regions indicate the interquartile range of the top 25 models, selected based on the training error, for species S and P respectively. The sampling duration used for training is marked in the grey shaded region. The ground truth model is also visualised with the black solid (S) and dashed (P) lines to allow comparison of the model fits. (TIF) [file pcbi.1012198.s003.tif]

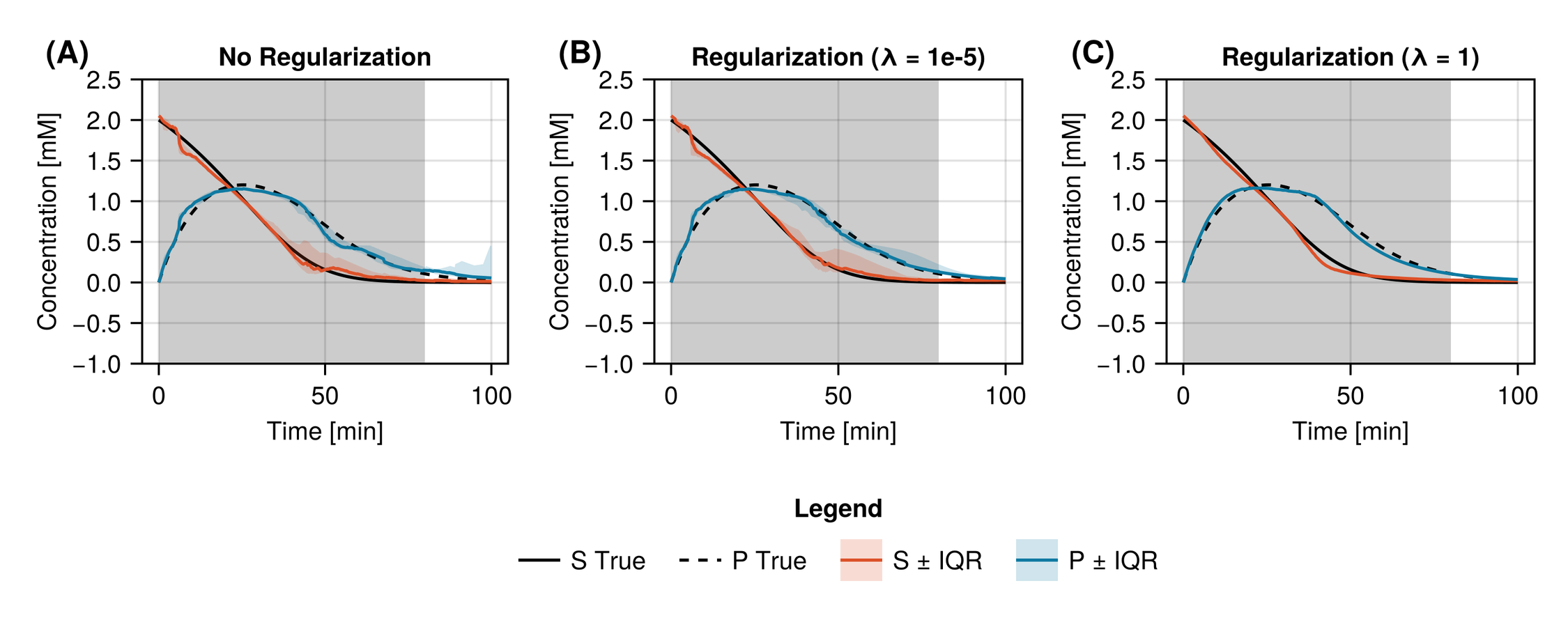

Supplement: S4 Fig — Median predictions for the Michealis-Menten model for species S (red) and species P (blue) including the first and third quartiles for A: the case without regularisation (λ = 0), B: mild regularisation (λ = 10-5), and C: strong regularisation (λ = 1) for a sampling duration of 80 minutes. The red and blue shaded regions indicate the interquartile range of the top 25 models, selected based on the training error, for species S and P respectively. The sampling duration used for training is marked in the grey shaded region. The ground truth model is also visualised with the black solid (S) and dashed (P) lines to allow comparison of the model fits. (TIF) [file pcbi.1012198.s004.tif]

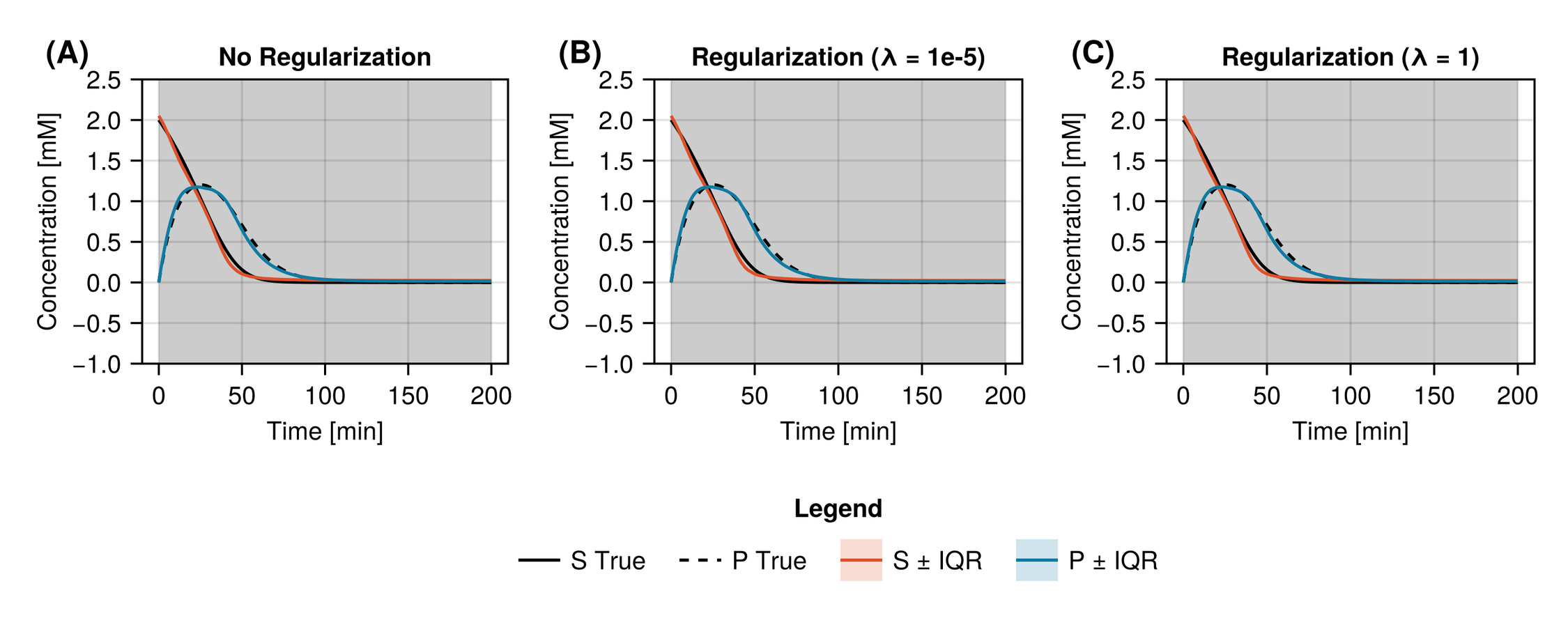

Supplement: S5 Fig — Median predictions for the Michealis-Menten model for species S (red) and species P (blue) including the first and third quartiles for A: the case without regularisation (λ = 0), B: mild regularisation (λ = 10-5), and C: strong regularisation (λ = 1) for a sampling duration of 200 minutes. The red and blue shaded regions indicate the interquartile range of the top 25 models, selected based on the training error, for species S and P respectively. The sampling duration used for training is marked in the grey shaded region. The ground truth model is also visualised with the black solid (S) and dashed (P) lines to allow comparison of the model fits. (TIF) [file pcbi.1012198.s005.tif]

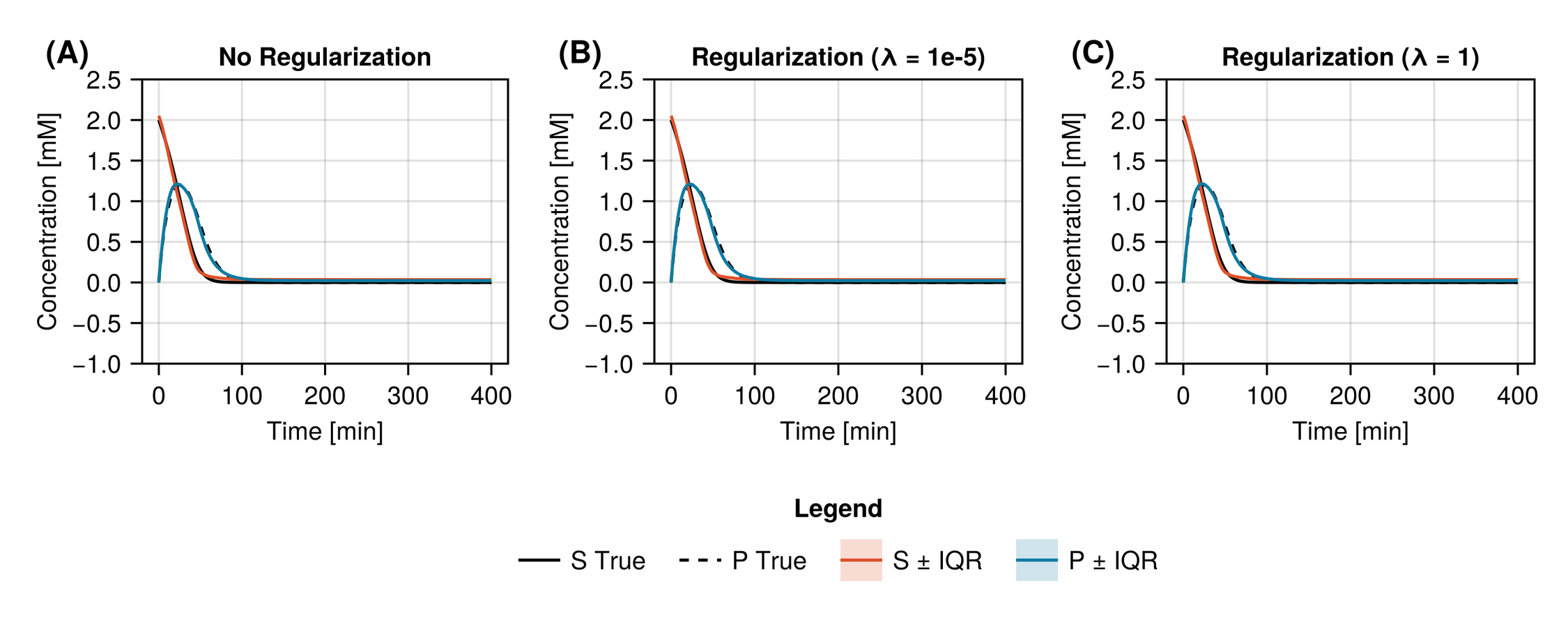

Supplement: S6 Fig — Median predictions for the Michealis-Menten model for species S (red) and species P (blue) including the first and third quartiles for A: the case without regularisation (λ = 0), B: mild regularisation (λ = 10-5), and C: strong regularisation (λ = 1) for a sampling duration of 400 minutes. The red and blue shaded regions indicate the interquartile range of the top 25 models, selected based on the training error, for species S and P respectively. The sampling duration used for training is marked in the grey shaded region. The ground truth model is also visualised with the black solid (S) and dashed (P) lines to allow comparison of the model fits. (TIF) [file pcbi.1012198.s006.tif]

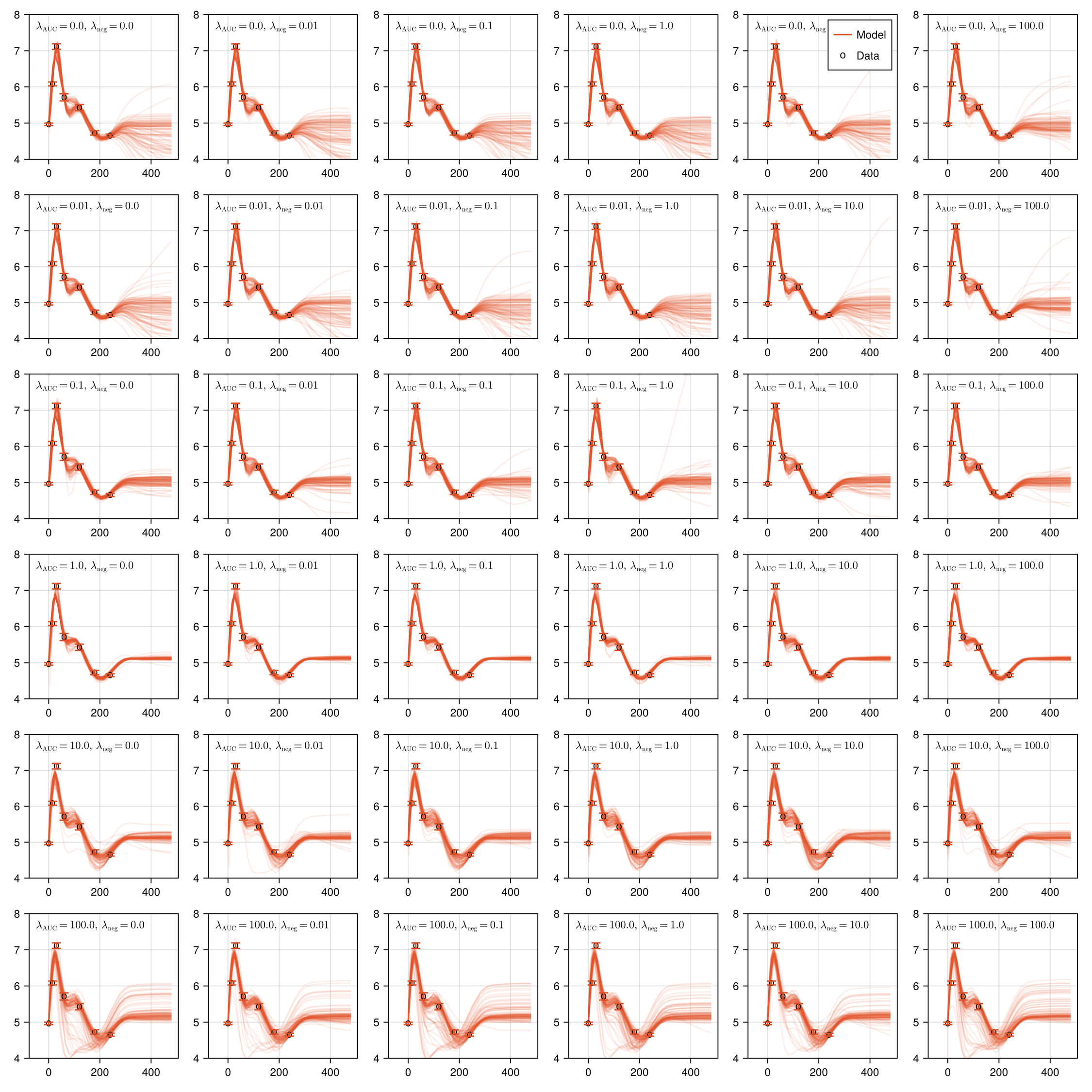

Supplement: S7 Fig — Each solid line represents a model with a unique initial parameter set. The data is shown in circles. Each column represents the non-negativity regularisation strength, while each row represents the area-under-curve regularisation strength. (TIF) [file pcbi.1012198.s007.tif]
