# Supplementary material for: Physiology-informed regularisation enables training of universal differential equation systems for biological applications
Source: PLoS Comput Biol. 2025 Jan 23;21(1):e1012198. doi: 10.1371/journal.pcbi.1012198 (PMC11771921; doi:10.1371/journal.pcbi.1012198)
Supplement: S1 Text — As an additional validation of the regularisation performance, the Michaelis-Menten model was extended by including an additional inhibitor molecule I. Using the more complicated model, the benefit of physiology-informed regularisation was evaluated. (PDF) [file pcbi.1012198.s008.pdf]

S5 Regularisation UDE in an extended Michaelis-Menten model with competitive inhibition.

As an additional validation of the regularisation performance, the Michaelis-Menten model was extended by including an additional inhibitor molecule $I(t)$. The resulting model equations for this model became:

$$\frac{dS(t)}{dt} = k_S S(t) - k_{SP} \frac{S(t)}{K_{M,app} + S(t)}$$

$$\frac{dP(t)}{dt} = k_{SP} \frac{S(t)}{K_{M,app} + S(t)} - k_P P(t)$$

$$\frac{dI(t)}{dt} = k_P P(t) - e_I I(t)$$

With

$$k_{M,app} = k_M \left(1 + \frac{I(t)}{k_I}\right)$$

In which the Michaelis-Menten term now additionally accounts for competitive inhibition. Using this model data was simulated for 200 minutes, with samples every 10 minutes. The used parameter values were $k_S = 9 \cdot 10^{-3}$, $k_{SP} = 0.2$, $k_M = 1.1$, $k_P = 0.08$, $k_I = 6 \cdot 10^{-2}$, and $e_I = 0.03$.

The Michaelis-Menten term with inhibition was replaced in full with a neural network of the same size as the original Michaelis-Menten experiment. The only difference is that the neural network now has three inputs, accounting for the additional state-variable in the model. The model was trained in the same way as the Michaelis-Menten model, with 50 initial parameters for regularisation strengths (λ) of 0, 10^{-5}, 10^{-3}, 1.0, and 10.0. From the resulting models, the top 25 models based on the validation error were selected. The mean and standard deviation of the model fits and forecasts are shown in figure S5.1.

This figure shows similar results to the original Michaelis-Menten model, where slight regularisation strongly improves the validation errors and corresponding model fits, while a too strong regularisation penalty then increases the validation error slightly again.

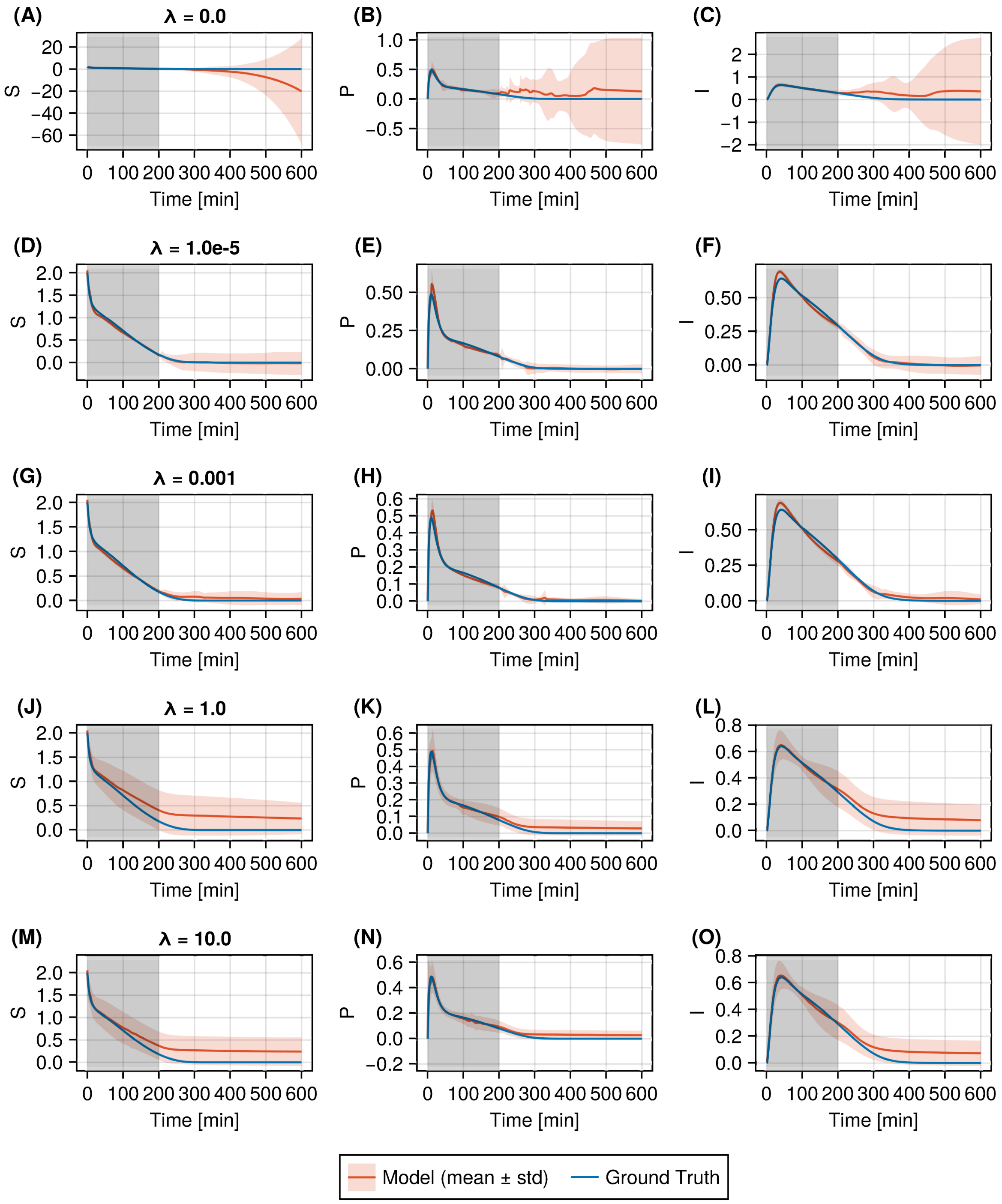

*Figure S5.1 **Visualization of learned Michealis-Menten Inhibition models trained with and without regularisation, on a sampling duration of 200 minutes, sampled every 10 minutes.** Mean and standard deviations of the species S, P, and I for regularization strengths $\lambda = 0$ (A-C), $\lambda = 10^{-5}$ (D-F), $\lambda = 10^{-3}$ (G-I), $\lambda = 1.0$ (J-L), $\lambda = 10.0$ (M-O). The solid orange line indicates the mean model fit and the red shaded area represents the standard deviation of the model fit. The blue line is the validation data, and the grey shaded area indicates the timepoints for which training data was provided.*
