# Supplementary material for: Physiology-informed regularisation enables training of universal differential equation systems for biological applications
Source: PLoS Comput Biol. 2025 Jan 23;21(1):e1012198. doi: 10.1371/journal.pcbi.1012198 (PMC11771921; doi:10.1371/journal.pcbi.1012198)
Supplement: S2 Text — As a rate-of-appearance function, a delayed pulse input was modelled, described by Korsbo et al. [33]. (PDF) [file pcbi.1012198.s009.pdf]

S8 Rate of Appearance

Given the absence of model equations to describe the rate of glucose appearance from the meal ($Ra(t)$), a delayed pulse input function was used to describe plasma glucose appearance for parameter estimation. This function is derived from a glucose pulse input function (dirac delta) subject to a linear chain of delays with a real-valued depth σ and a rate parameter k_{meal}, which is assumed to be constant across each delay compartment. Assuming that the initial meal input can be modelled using a Dirac delta, the resulting delayed glucose input is formulated as: [1]

$$Ra(t) = \frac{1}{\Gamma(\sigma)} (k_{meal})^{\sigma} \cdot t^{\sigma-1} \exp(-k_{meal} \cdot t)$$

Where $\Gamma(\cdot)$ denotes the gamma function. For σ and k_{meal}, the values 1.4 [-] and 0.014 [min^{-1}] were used, respsectively.

Bibliography

[1] N. Korsbo and H. Jönsson, "It's about time: Analysing simplifying assumptions for modelling multi-step pathways in systems biology," *PLOS Comput. Biol.*, vol. 16, no. 6, p. e1007982, Jun. 2020, doi: 10.1371/journal.pcbi.1007982.
