# Supplementary material for: Physiology-informed regularisation enables training of universal differential equation systems for biological applications
Source: PLoS Comput Biol. 2025 Jan 23;21(1):e1012198. doi: 10.1371/journal.pcbi.1012198 (PMC11771921; doi:10.1371/journal.pcbi.1012198)
Supplement: Attachment — Submitted filename: response_to_reviewers.pdf [file pcbi.1012198.s011.pdf]

Manuscript PCOMPBIOL-D-24-00878
Response to reviewers

Reviewers' comments to the authors:

Reviewer 1

The manuscript presents and evaluates a method to regularize universal differential equations (UDE) to avoid or at least limit unrealistic solutions. The approach is tested using two well-known examples in Systems Biology: the case of an enzyme catalysed reaction with Michaelis-Menten kinetics and blood glucose levels following ingestion of a meal.

Appearance of negative concentrations is a well known issue of ODE's when used in Systems Biology and the proposed approach appears to limit those non-biological/non-realistic cases very efficiently when the approach is extended to UDEs. In the case of blood glucose levels the regularization approach also seems to accomplish the fact that all ingested glucose eventually appears in blood (and does not disappear in the gastrointestinal tract).

Universal differential equations are a promising approach to combine the advantages of knowledge based models built using differential equations and the potential of machine learning (neural networks). This method tests, illustrates and expands this work and it is a useful addition to the field.

<u>Comment #1</u>
Code and code availability and accessibility: the code has been made available in git. The code is reasonably well structured and it is possible to run the examples. Dependencies are not clearly stated in the Readme file. Please list the required dependencies for each example so that the code can be tested.

Response: Thank you for pointing out that the required dependencies were not stated clearly. While the dependencies were listed in the 'Project.toml' file, this was not mentioned in the Readme file. The Readme file has been updated, such that it explicitly refers to the 'Project.toml' file, listing the dependencies of the code. Additionally, the specific versions of each package have been added to the [compat] section of the 'Project.toml' file to ensure reproducibility.

<u>Comment #2</u>

The code is not commented, please add comments so that the code can be easily read, understood and adapted. Add descriptive comments to the functions to clarify what the inputs and outpus are. Also add inline comments to describe what the code is intended to produce and what the variables denote. For example, reading the function michaelismenten_ude it is not directly evident to know which A or B is the product or the substrate.

Response: We acknowledge that the amount of documentation in the code was limited. We have now added docstrings to all functions that have been defined, explaining their use. We have changed the variable names for the state variables in the michaelismenten_ude function from A and B, to S and P, to be consistent with the manuscript. Furthermore, we have checked all other functions and scripts and added additional comments where necessary, to clarify the code. We believe these changes will improve the overall readability of the code and aid in its re-use by other researchers.

<u>Comment #3</u>

Materials and Methods:
Notation and explanations of the equations can largely be improved by using more accurate and concise mathematical notation. The vector variables are usually denoted with bold font (or and "arrow " on top of the variable). This is done here inconsistently. For example in equation 1 it is clear that u denotes a vector, but the p and \theta (sets of parameters) are not considered vectors but scalars. Also equations 2 and 3 use bold font to denote S and P (which are scalars…). Similarly G and X in equations 8 and 9 should not be in bold font. Please check everywhere and ensure consistent use of bold to denote vectors.

Response: We have changed the notation of equations, such that all bold font variables denote vectors, and all regular weight font variables denote scalars.

Comment #4

Also from equations 2 and 3 it appears that a vector u is defined that is used in later on (equations 5 to 7).. however this vector is never explicitly defined.

Response: We have clarified the definitions of vectors **u** that we have used in the equations (156-159).

Comment #5

Finally the use of A and B as state variables line 149 appears to be a mistake that should be corrected (even though it does link with the code files).

Response: Thank you for pointing this out. We have now changed A and B to S and P to be consistent with the rest of the manuscript. (line 147)

Comment #6

The lambda values provided in line 166 appear incomplete, as in the text a lambda value of 1 is often cited, (that should match to x=0, not included in the range).

Response: We have added the lambda value of 1 to the list. (line 179)

Comment #7

Please include in the text the regularization penalty that is included to ensure the glucose area under the curve is one. This is an important function that should be explicitly included.

Response: The equation and implementation details on the regularization penalty were added to the main text. (lines 235-239)

Comment #8

Please double check the precision and accuracy of the mathematical descriptions in the materials and methods section.

Response: We have additionally rechecked the mathematical descriptions in the materials and methods section of the paper and can confirm that the incorrect expressions have now been corrected.

Results

Comment #9

The authors compared the performance of the UDE system with and without the regularization function. However they do not compare to an ODE only model in both cases. The authors should briefly comment in which cases using UDE outperforms the use of ODE models for the applications here presented.

Response: As the data for the Michealis Menten model was generated using the full ODE model, in this instance we expect that the ODE would perform as well or better than the UDE.Consequently, we decided not to include a comparison to the complete ODE model. In this study, we do not propose that UDEs would out-preform a well-validated ODE model. UDEs are beneficial for gap filling, namely learning currently unknown components of an existing ODE models, as simulated in our study by infering the rate of conversion between metabolites S and P in the Michealis-Menten model and the rate of glucose appearance in the Bergman minimal model. In instances where the systems is known, and a well validated ODE model is available would always advocate for the use of the ODE model. We have now clarified this use case of a UDE model compared to an ODE model in the discussion (lines 406-409)

Comment #10

The models are set to be simulated in real life settings with 5% noise. To what extent is this 5% a reasonable representation of the noise in these systems? How would the approach work with higher (10% , 15%) noise levels.

Response: The level of 5% noise was chosen matching with previous work done on universal differential equations. [1] Furthermore, typical clinical measurements of glucose express errors below 5%. Nevertheless, we agree that it is relevant to test the UDE model with regularization in a situation with more noise. We additionally performed the simulations depicted in Fig. 2 A and B with a minimum noise level of 10%, showing a similar result to the simulation with 5% noise. A figure of those results has been added to supplementary material S1 Fig. Like any data-driven approach, UDEs are sensitive to the quality

of the data. In situations with very high noise, the UDE model may fail to converge, or converge towards an erroneous solution. We have additionally added this to the discussion (lines 397-400).

Comment #11

Also a typical characteristic of the considered problems is the appearance of missing data points. How would the method perform if data points are missing from the time series?

Response: We agree with the reviewer that missing data is a relevant scenario to take into account for the considered problems. As a data driven approach , UDEs are naturally sensitive to the quality of the data available and will be vulnerable to missing data. In Figure 4, we evaluate the effect of different sampling frequences and durations on the quality of the model fits and the impact of the physiology-based regularisation. Up to the amount of time between samples investigated, we have found that the duration of sampling has a larger effect on the eventual validation error, as this impacts the steady-state behaviour of the model. With more frequently sampled data or with longer sampling duration, the UDE can better approximate the true dynamics, even without the addition of physiology-informed regularisation. For sparser are shorter sampling frequencies, the benefit of the addition of physiology-based regularisation is shown, guiding the UDE towards more biologically plausible behaviours. These sparser sampling schedules would be representative of missing data scenarios. However, the overall performance of UDEs is highly dependent on the quality of the data available, if many time points are missing, or if data points around crucial systems dynamics, the UDEs will be very vulnerable to this. However, we also face these challenges when fitting ODE systems to time series data. This has been clarified in the discussion. (lines 393-397)

Comment #12

Figures 2 and 3 provide results corresponding to a sampling duration of 40 min every 5min. To what extent are the results relevant for other sampling scenarios? In addition to the data in Figure 4 the authors should indicate whether the observed improvements also hold for more extreme sampling cases.

Response: Thank you for pointing out that the performance of the UDE model with regularization in extreme sampling cases was not clearly shown. We have added additional examples for extreme sampling cases to the supplementary material S3 Fig., and S4 Fig. (Figure 1 and Figure 2) and clarified that the effect of regularization additionally depends on the quality of available data (lines 262 – 265)

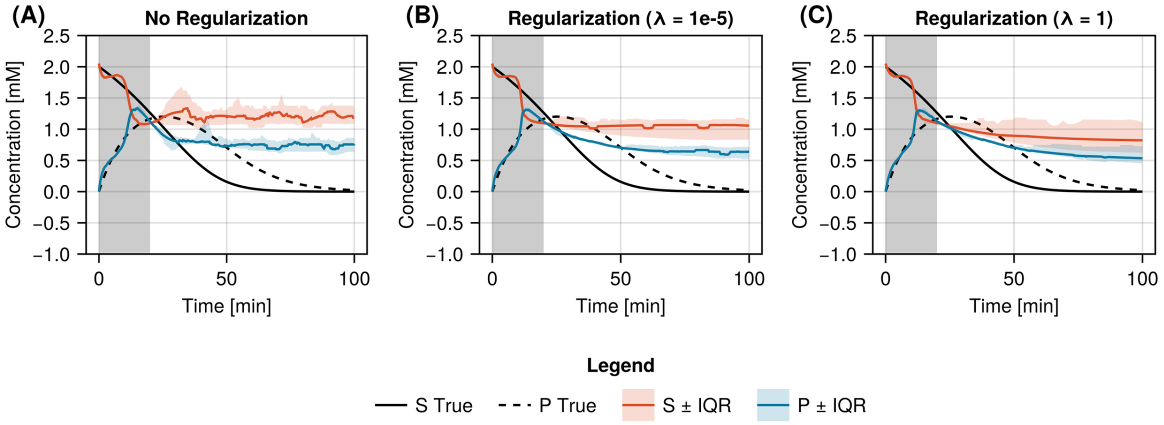

Figure 1.

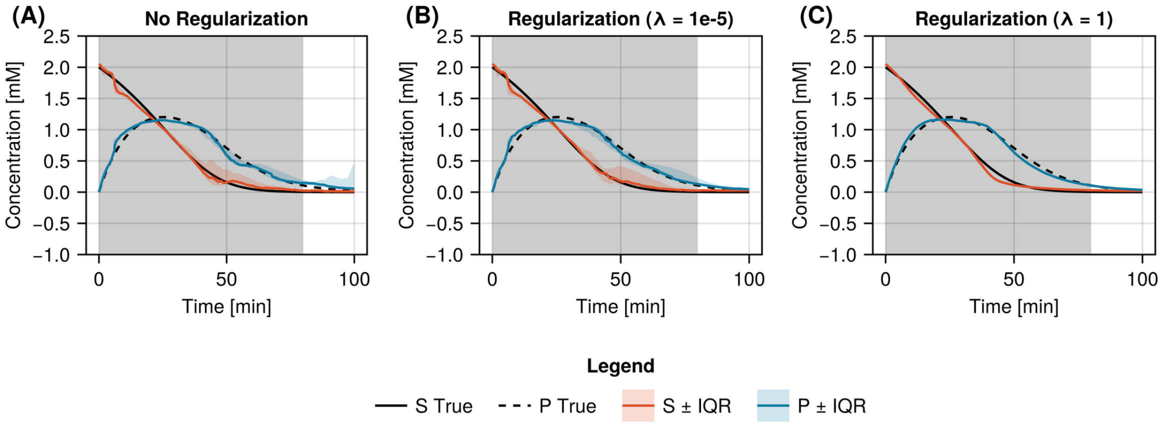

Figure 2

<u>Comment #13</u>

Figure 3 legend: why is it stated (\lambda > 0.1 as a criterion for there being a regularization? Is this a typo?

Response: The typo has been removed. Thank you for pointing this out.

<u>Comment #14</u>

Figure 4. When comparing the effect of different sampling frequencies it appears that sampling every 20 minutes leads to lower error than sampling every 5 or 10 minutes (at least for lambda=0). Can the authors comment on this? Also what happens if very long sampling durations are considered (up to 400 minutes?)

Response: We agree that the lower error observed for the sparse sampling frequency is unexpected. Having taken a deeper exploration of the underlying data we found that this discrepancy stems from the models with 20 minutes having a higher likelihood of learning a steady-state, whereas the models with sampling every 5 minutes receive more data points which all also have noise. These 5-minute models are potentially more influenced by noise than the 20-minute models, particularly for shorter sampling durations, and the learned behaviour often tend to explode beyond the sampling duration, not finding a steady-state. While the steady-state learned by the model trained on samples every 20 minutes is not correct, the two unwanted results do show differences in their error values. We have provided additional explanation of this phenomenon in the discussion (lines 356-362). Furthermore, we found that the code for the original figure did not calculate the standard deviations correctly. Therefore, we have updated this figure to show the correct standard deviations. (Fig.4 in the manuscript, see Figure 3 below)

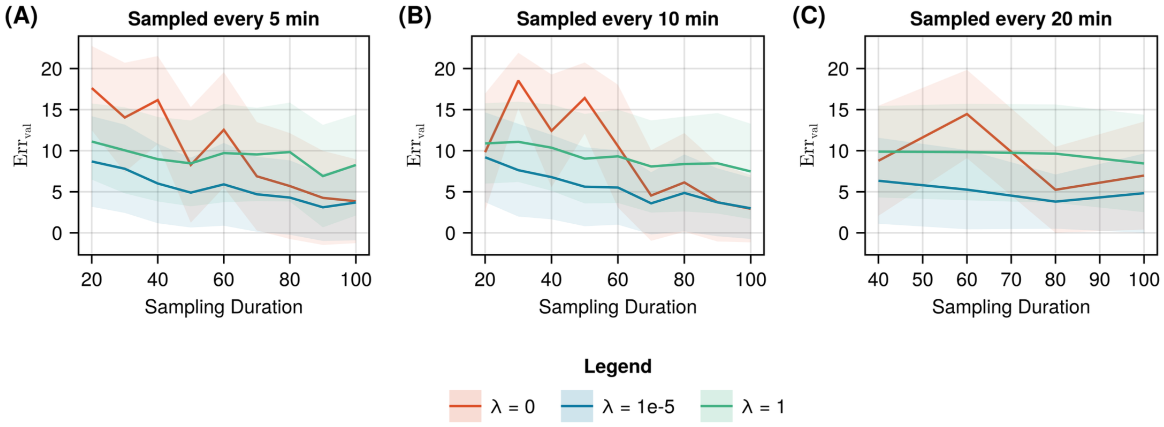

Figure 3

Comment #15

Figure 5 shows a clear difference between the regularized and the non regularized models. From the two regularization terms introduced (negative concentrations, area under the curve) what is the impact of each one? Is this effect due to the combination of both terms or can it be attributed to only one of them? From 5a and 5c it seems that the appearance of negative G/X is not happening often, so that regularization should not have a major effect. Can the authors evaluate the relative impact of each contribution?

Response: We agree that the contribution of the individual regularization terms is not clearly described. In supplementary figure S9 Fig., it can be seen that the nonnegativity penalty reduces the model producing a downward trend after all data has been seen, while the area under the curve penalty further stabilises the model simulations. An additional benefit of using both penalties combined is that the nonnegativity penalty prevents the model from compensating for the area under the curve penalty by predicting a negative rate of appearance. We have added an additional paragraph to the discussion, highlighting the individual effects of both regularization terms (lines 369-375)

Comment #16

Typos and Mistakes: please check the spelling of Michaelis-Menten (there are multiple cases of Michealis-Menten) that should always be written with upper case letters. (Same for Gaussian)

Response: Thank you for highlighting these errors, We have now reviewed the entire manuscript and the spelling of Michaelis-Menten and Gaussian have been checked and adjusted where necessary.

Reviewer 2

Regularization methods for generalizing Neural Networks for learning UDE parameters with real and simulated data are presented by the authors. Although, the approach and results presented are limited to a simple two state model, details of the approach and limitations are well documented

Comment #1

Line 374: In summary, we have presented physiology-informed regularization as a simple, yet powerful and generalizable approach for the improvement of UDE training in biological models. The used of physiology-informed regularization not only improves long-term predictive stability, reducing model variance, but it can also be seen to reduce non-physiological behavior in the neural network component.

Response: Thank you for pointing out the mistakes in these sentences. We have updated the sentences according to your suggestions.

Reviewer 3

In this manuscript, the authors use two physiology informed regularization terms to help identify better weights of the neural network component of a UDE model. UDE models combine mechanistic differential equations that describe known components of a model with neural networks to describe unknown components of a model. A current limitation to using UDE models to describe biological systems is the sparse data common to biological applications. To address this limitation, the authors implement this physiology informed regularization approach to train UDE models of two simple example systems, 1. simulated data from a Michaelis-Menten model of a single enzyme reaction, and 2. average measured human plasma glucose and insulin concentrations at 7 time points. The authors demonstrate how adding regularization terms including one that penalizes negative metabolite concentrations, can improve the predictiveness of the UDE models using less training data than if no regularization terms are used.

Comment #1

The UDE model construction/formulation could be clearer. More description and an additional figure that shows the connection between the differential model and the neural network (NN), including what the inputs and output variables of the NN would help (for both the Michaelis-Menten (MM) model and the glucose model). For the MM model, I think the inputs are the S and P concentrations and the output is the flux of the MM reaction? For the glucose model, I think the output is the plasma glucose rate appearance, Ra, but I'm not sure what the input variable is to the NN.

Response: We have extended the explanation of the neural network structure for both models. For the Michaelis-Menten model, we have explicitly mentioned the inputs (S(t) and P(t)) and the output of the neural network, which is the flux of the Michaelis-Menten reaction (lines 144-147). Similarly, for the minimal model, we have specified the input (t) (line 228). Additionally, Figure 1 and its description has been updated (see Figure 4) to clarify the inputs and outputs of the neural network model.

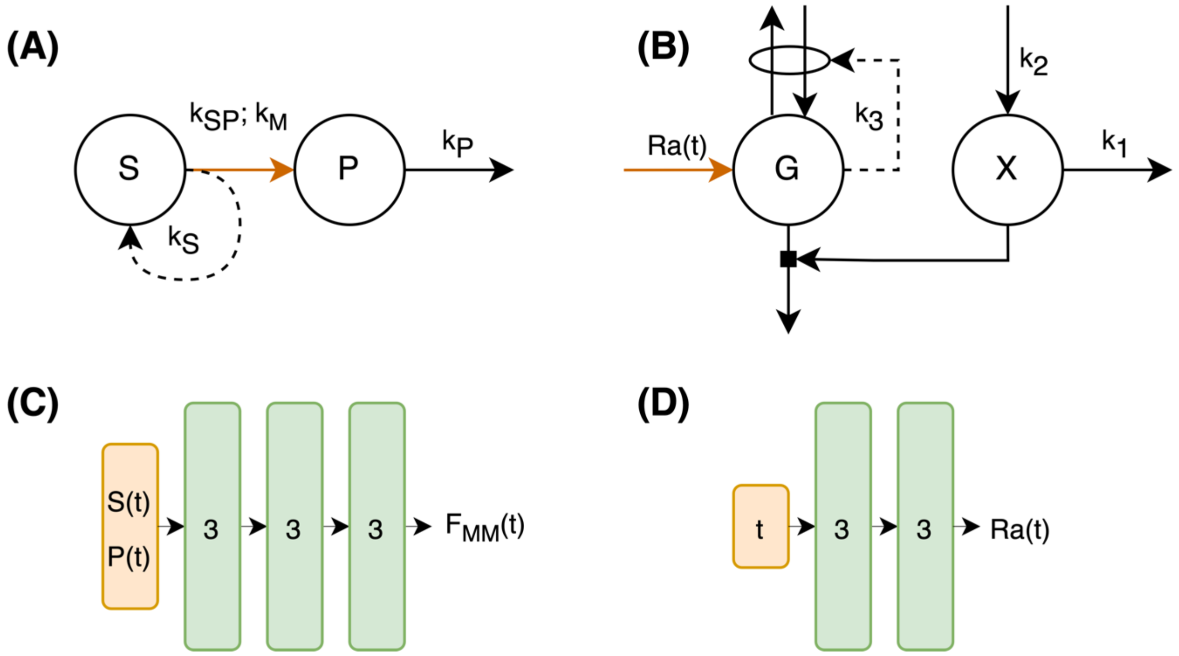

Figure 4

Comment #2

How was the NN architecture/structure determined for the two models?

Response: For the Michaelis-Menten model, a grid search of number of layers and number of nodes per layer was used. The model with the lowest average validation error on the data of 100 minutes of sampling, sampled every 5 minutes, was taken. This has been clarified in the text of the methods. (lines 147-150) For the Minimal-Model, the same neural network structure was used, but we decreased the depth of the model to 2, reducing its complexity. (lines 226-230)

Comment #3

I think this paper would be strengthened with some additional analyses with the MM model and adding an additional example with slightly more complicated MM models. For example,

How does the UDE model handle different initial S and P concentrations?

Response: We agree that this is a very relevant aspect in the case of universal differential equations. After training our neural network on the initial data, we changed the initial conditions of the model and resimulated the UDE model with the trained neural network and compared it to the ground truth model with the same initial conditions. From this analysis, we can see that the UDE model handles different S and P concentrations similarly to the data with the same initial conditions. We have added an additional figure to the supplementary material (S2 Fig., see Figure 5), showing the forecasts of the neural network when simulating new data with different initial S and P concentrations. (lines 293-295)

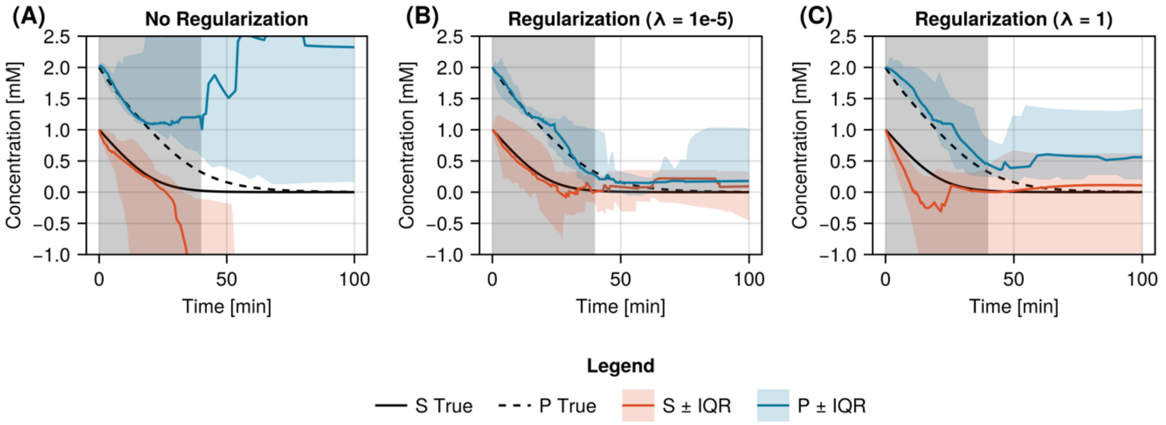

Figure 5

Comment #4

Can the UDE with physiological regularization capture more complicated MM reactions, such as those that involve inhibitions?

Response: We agree that the investigation of the ability of a universal differential equation to recover a more complicated term is relevant to the field in general. To complement the analysis of the Michaelis-Menten kinetics, we have additionally tested a scenario using a slightly more complicated model, involving competitive inhibition. The more complicated model has the equations

$$\frac{dS}{dt} = k_S S - k_{SP}\frac{S}{k_{M,app} + S}$$

$$\frac{dP}{dt} = k_{SP}\frac{S}{k_{M,app} + S} - k_P P$$

$$\frac{dI}{dt} = k_P P - e_I I$$

With

$$k_{M,app} = k_M\left(1 + \frac{I}{k_I}\right)$$

Using this model data was simulated for 200 minutes, with samples every 10 minutes. The used parameter values were kS = 9 * 10^(-3), kSP = 0.2, kM = 1.1, kP = 0.08, kI = 6 * 10^(-2), and eI = 0.03. The Michaelis-Menten term with inhibition was replaced in full with a neural network of the same size as the original Michaelis-Menten experiment. The only difference is that the neural network now has three inputs, accounting for the additional state-variable in the model. The model was trained in the same way as the Michaelis-Menten model, with 50 initial parameters for regularization strengths of 0, 10^(-5), 0.001, 1.0, and 10.0. From the resulting models, the top 25 models based on the validation error were selected. The results of this experiment can be seen below (Figure 6), which shows the mean and standard deviation model fits and forecasts for each state variable (S, P, and I) and regularization strength, together with the validation data for each of the state variables.

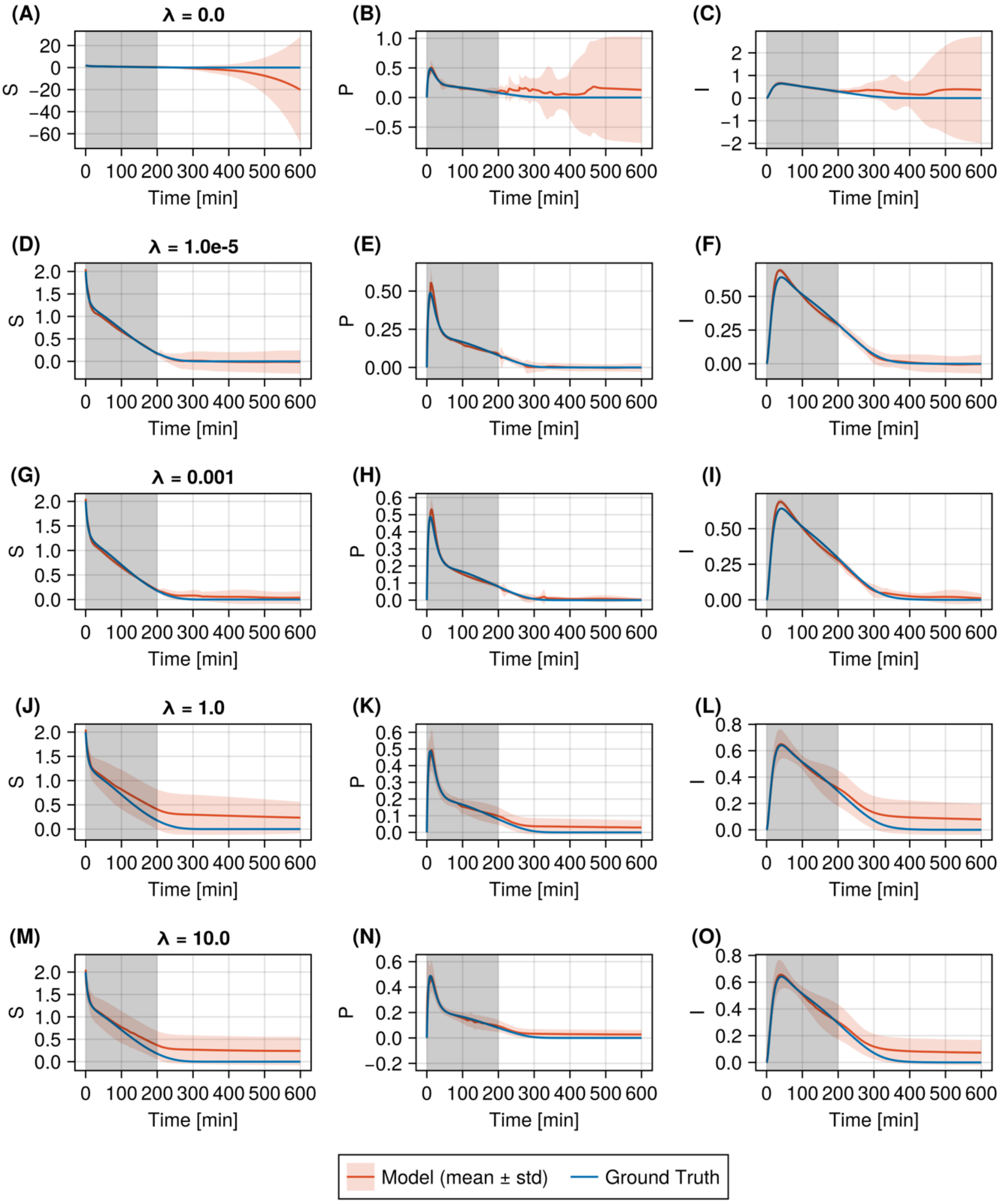

Figure 6

This figure shows similar results to the original Michaelis-Menten model, where slight regularization strongly improves the validation errors and corresponding model fits, while a too strong regularisation penalty then increases the validation error slightly again. We have added this experiment to the supplementary material (supplementary material S5) and refer to it in the results (lines 276-277) and discussion. (lines 402-404)

This example shows that the regularization benefit is not limited to simple kinetic terms, but can also be applied in slightly larger, and more complicated models. Yet, the successful recovery of equations using a neural network additionally depends on data availability. As neural networks are universal

approximators, given sufficient data, they will theoretically be able to describe any relationship in the data.

Comment #5

How would a somewhat larger model perform? Is there a computational limitation to using this UDE method with larger models?

Response: In our response to comment #4 we have added an additional example with a slightly larger model. Whereas a UDE is computationally more demanding compared to a regular ordinary differential equation model, the availablity data typically limits the size of the neural network that can be trained. We have added this additional explanation to the discussion. (lines 400-402) Consequently, the NN used in UDE models do not tend to be excessively large. Furthermore, the use of the Julia programming language can strongly increase the computational efficiency of the code Additionally, the implementation of fast computation of gradients using packages from the SciML ecosystem strongly increases the scalability of UDE models.

Comment #6

How does this approach fit into the broader space of Biology/Physics/Physiology Informed Neural Networks?

Response: Physics-Informed Neural Networks (PINNs) are another approach to combining neural networks with differential equations. Where in a UDE, the neural network is a part of the differential equation system, in PINNs, the neural network is used to directly learn thedynamic behaviour of the complete system and the error function is regularized by a known system of differential equations, to ensure the found solution adheres to assumedphysical laws. Potentially, physiology-informed regularization presented in this study could also be beneficial for PINNs , but this would warrant further investigation. We have included this brief discussion of physics-informed neural networks in the discussion (lines 442-452)

Comment #7

Lines 243-251, Fig 3 and the Fig 3 caption: The text in these lines and the caption text are not consistent, and in some cases do not match what is shown in the figure. In the caption for Fig 3, you state "The incorporation of regularization ($\lambda \geq 0.1$) during the training of the Michealis-Menten UDE improves the long-term dynamics of the learned model terms with the majority of validation errors located in the vicinity of 4 Errval." And in lines 250-251 you have "For stronger regularization strengths, the peak around Errval = 4 becomes sharper." Which does not match what is shown in Fig 3.

The last sentence of your caption, "The effect of the regularization strength on the performance is mainly seen in the size of the peak around Errval = 4, which becomes larger for stronger until $\lambda = 10-5$ and then flattens out, as more model fits remain at larger validation error values," seems to describe more accurately what is shown in Figure 3.

Response: Thank you for pointing this out, the caption of figure 3 has now been adjusted such that it matches the figure.

Comment #8

Line 103 (and elsewhere): The section heading "Regularization in a simulated model of Michaelis-Menten kinetics," is confusing. I think you are referring to the fact you are using simulated data to train and validate your UDE model, but that is not clear from the heading.

Response: We have changed the heading such that it is now clearer that we are using simulated data to train and validate the UDE model.

Comment #9

Lines 104-106: This sentence is confusing.

Response: The sentence has been clarified.

Comment #10

Lines 103, 134 and maybe elsewhere: Michaelis-Menten should be capitalized.

Response: Michaelis-Menten has been capitalized all through the manuscript.

Comment #11

Fig 1C and line 136: The text says there is a single output, but the figure shows 2 outputs (2xn). Should the figure show 1xn for the output?

Response: Figure 1 has been adjusted. The neural network in the figure now shows only one output.

Comment #12

Line 149: you refer to state variables A or B, but this is the only place you use A or B as variable names. You should either replace with S and P or include a general equation that represents your UDE system using A and B as state variables, and then make it clear what A and B are in both of the MM and Glucose models.

Response: A and B have been changed into S and P respectively.

Comment #13

Line 151: "grows linearly with the square of their magnitudes." It would be clearer to say "grows quadratically."

Response: Thank you for the suggestion, twe have adopted this term in line 151.

Comment #14

You refer to the glucose model as Bergman's minimal model, glucose minimal model, glucose oral minimal model, and minimal model at different points throughout the manuscript. It would be clearer if you used a consistent name throughout.

Response: We have changed our referring to the glucose model as the glucose minimal model consistently.

Comment #15

Line 249, Fig 3 caption, maybe elsewhere: You interchangeably use "Eval" and "Errval". Additionally, "4 Errval" should be "Errval = 4"

Response: We have changed Eval to Errval, and changed "4 Errval" to "Errval = 4".

Comment #16

Line 279: "over a range of 1 mmol / L around the fasting plasma glucose" is a little confusing and could be misinterpreted as more than a 1mmol / L difference. I'd suggest something like "within ± 0.5 mmol / L of the fasting plasma glucose."

Response: The suggestion has been followed.

Comment #17

Line 314: “with fewer information” should be “with less information”

Response: Thank you, we have updated line 314.
