# Supplementary material for: Physiology-informed regularisation enables training of universal differential equation systems for biological applications
Source: PLoS Comput Biol. 2025 Jan 23;21(1):e1012198. doi: 10.1371/journal.pcbi.1012198 (PMC11771921; doi:10.1371/journal.pcbi.1012198)
Supplement: Attachment — Submitted filename: Comments.docx [file pcbi.1012198.s010.docx]

In this manuscript, the authors use two physiology informed regularization terms to help identify better weights of the neural network component of a UDE model. UDE models combine mechanistic differential equations that describe known components of a model with neural networks to describe unknown components of a model. A current limitation to using UDE models to describe biological systems is the sparse data common to biological applications. To address this limitation, the authors implement this physiology informed regularization approach to train UDE models of two simple example systems, 1. simulated data from a Michaelis-Menten model of a single enzyme reaction, and 2. average measured human plasma glucose and insulin concentrations at 7 time points. The authors demonstrate how adding regularization terms including one that penalizes negative metabolite concentrations, can improve the predictiveness of the UDE models using less training data than if no regularization terms are used.

Comments:

1. The UDE model construction/formulation could be clearer. More description and an additional figure that shows the connection between the differential model and the neural network (NN), including what the inputs and output variables of the NN would help (for both the Michaelis-Menten (MM) model and the glucose model). For the MM model, I think the inputs are the S and P concentrations and the output is the flux of the MM reaction? For the glucose model, I think the output is the plasma glucose rate appearance, Ra, but I’m not sure what the input variable is to the NN.

How was the NN architecture/structure determined for the two models?

2. I think this paper would be strengthened with some additional analyses with the MM model and adding an additional example with slightly more complicated MM models. For example,

- How does the UDE model handle different initial S and P concentrations?
- Can the UDE with physiological regularization capture more complicated MM reactions, such as those that involve inhibitions?
- How would a somewhat larger model perform? Is there a computational limitation to using this UDE method with larger models?

3. How does this approach fit into the broader space of Biology/Physics/Physiology Informed Neural Networks?
4. Lines 243-251, Fig 3 and the Fig 3 caption: The text in these lines and the caption text are not consistent, and in some cases do not match what is shown in the figure. In the caption for Fig 3, you state “The incorporation of regularization (λ ≥ 0.1) during the training of the Michealis-Menten UDE improves the long-term dynamics of the learned model terms with the majority of validation errors located in the vicinity of 4 Errval.” And in lines 250-251 you have “For stronger regularization strengths, the peak around Errval = 4 becomes sharper.” Which does not match what is shown in Fig 3.

The last sentence of your caption, “The effect of the regularization strength on the performance is mainly seen in the size of the peak around Errval = 4, which becomes larger for stronger until λ = 10−5 and then flattens out, as more model fits remain at larger validation error values,” seems to describe more accurately what is shown in Figure 3.

Minor Comments

1. Line 103 (and elsewhere): The section heading “Regularization in a simulated model of Michaelis-Menten kinetics,” is confusing. I think you are referring to the fact you are using simulated data to train and validate your UDE model, but that is not clear from the heading.
2. Lines 104-106: This sentence is confusing.
3. Lines 103, 134 and maybe elsewhere: Michaelis-Menten should be capitalized.
4. Fig 1C and line 136: The text says there is a single output, but the figure shows 2 outputs (2xn). Should the figure show 1xn for the output?
5. Line 149: you refer to state variables A or B, but this is the only place you use A or B as variable names. You should either replace with S and P or include a general equation that represents your UDE system using A and B as state variables, and then make it clear what A and B are in both of the MM and Glucose models.
6. Line 151: “grows linearly with the square of their magnitudes.” It would be clearer to say “grows quadratically.”
7. You refer to the glucose model as Bergman’s minimal model, glucose minimal model, glucose oral minimal model, and minimal model at different points throughout the manuscript. It would be clearer if you used a consistent name throughout.
8. Line 249, Fig 3 caption, maybe elsewhere: You interchangeably use “Eval” and “Errval”. Additionally, “4 Errval” should be “Errval = 4”
9. Line 279: “over a range of 1 mmol / L around the fasting plasma glucose” is a little confusing and could be misinterpreted as more than a 1mmol / L difference. I’d suggest something like “within ± 0.5 mmol / L of the fasting plasma glucose.”
10. Line 314: “with fewer information” should be “with less information”
